# An epilepsy classification based on FFT and fully convolutional neural network nested LSTM

**DOI:** 10.3389/fnins.2024.1436619

**Published:** 2024-07-30

**Authors:** Jianhao Nie, Huazhong Shu, Fuzhi Wu

**Affiliations:** Laboratory of Image Science and Technology, Key Laboratory of Computer Network and Information Integration, Ministry of Education, Southeast University, Nanjing, China

**Keywords:** electroencephalogram, fast Fourier transformation, seizure detection, convolutional neural network, long-short term memory

## Abstract

**Background and objective:**

Epilepsy, which is associated with neuronal damage and functional decline, typically presents patients with numerous challenges in their daily lives. An early diagnosis plays a crucial role in managing the condition and alleviating the patients’ suffering. Electroencephalogram (EEG)-based approaches are commonly employed for diagnosing epilepsy due to their effectiveness and non-invasiveness. In this study, a classification method is proposed that use fast Fourier Transform (FFT) extraction in conjunction with convolutional neural networks (CNN) and long short-term memory (LSTM) models.

**Methods:**

Most methods use traditional frameworks to classify epilepsy, we propose a new approach to this problem by extracting features from the source data and then feeding them into a network for training and recognition. It preprocesses the source data into training and validation data and then uses CNN and LSTM to classify the style of the data.

**Results:**

Upon analyzing a public test dataset, the top-performing features in the fully CNN nested LSTM model for epilepsy classification are FFT features among three types of features. Notably, all conducted experiments yielded high accuracy rates, with values exceeding 96% for accuracy, 93% for sensitivity, and 96% for specificity. These results are further benchmarked against current methodologies, showcasing consistent and robust performance across all trials. Our approach consistently achieves an accuracy rate surpassing 97.00%, with values ranging from 97.95 to 99.83% in individual experiments. Particularly noteworthy is the superior accuracy of our method in the AB versus (vs.) CDE comparison, registering at 99.06%.

**Conclusion:**

Our method exhibits precise classification abilities distinguishing between epileptic and non-epileptic individuals, irrespective of whether the participant’s eyes are closed or open. Furthermore, our technique shows remarkable performance in effectively categorizing epilepsy type, distinguishing between epileptic ictal and interictal states versus non-epileptic conditions. An inherent advantage of our automated classification approach is its capability to disregard EEG data acquired during states of eye closure or eye-opening. Such innovation holds promise for real-world applications, potentially aiding medical professionals in diagnosing epilepsy more efficiently.

## 1 Introduction

Epilepsy is a very common neurological disorder in humankind that affects roughly 50 million people worldwide (Tuncer et al., 2021; World Health Organization, 2021). It is characterized by abnormal electrical activity in the nerve cells of the brain, resulting in recurrent seizures, unusual behavior, and possibly loss of consciousness (Fisher et al., 2014; Ozdemir et al., 2021). The worst-case scenario could result in permanent harm to the patient’s life. Up to 70% of individuals with epilepsy could live seizure-free if properly diagnosed and treated. Therefore, a timely and accurate diagnosis method for epilepsy is essential for all patients and doctors. In clinical practice, doctors diagnose epilepsy by using patients’ medical records, conducting neurological examinations, and employing various clinical tools such as neuroimaging and EEG recording. However, this analysis is considered complex due to the presence of patterns in the EEG that can be challenging to interpret, even for experienced experts. This complexity can lead to different opinions among experts regarding EEG findings, necessitating complementary examinations (Oliva and Rosa, 2019; Oliva and Rosa, 2021). To address the time-consuming nature of visual analysis and errors caused by visual fatigue during the increasing continuous EEG video recordings, numerous automatic methods have been developed.

There have been various methods proposed in the past three decades for the automatic identification of epileptic EEG signals (Ghosh-Dastidar and Adeli, 2009; Sharma et al., 2014; Shanir et al., 2018; Truong et al., 2018). Machine learning (ML) methods can be used to build effective classifiers for automatic epilepsy detection. These automatic seizure detection methods mainly include two steps: feature extraction and classifier construction. The feature extraction includes time domain (T) (Jaiswal and Banka, 2017; Gao et al., 2020; Wijayanto et al., 2020), frequency domain (F) (Altaf and Yoo, 2015; Kaleem et al., 2018; Singh et al., 2020), time-frequency domain (TF) (Tzallas et al., 2007; Abualsaud et al., 2015; Feng et al., 2017; Shen et al., 2017; Goksu, 2018; Sikdar et al., 2018; Yavuz et al., 2018), and a combination of nonlinear approaches (Zeng et al., 2016; Ren and Han, 2019; Sayeed et al., 2019; Wu et al., 2019). In addition, various types of entropy such as fuzzy entropy (Xiang et al., 2015), approximate entropy, sample entropy, and phase entropy (Acharya et al., 2012) have been calculated from the EEG signals to distinguish different epileptic EEG segments. The automatic seizure classifier includes Support Vector Machine (SVM) (Subasi and Ismail Gursoy, 2010; Das et al., 2016; Şengür et al., 2016; Li and Chen, 2021), Convolutional Neural Network (CNN) (Feng et al., 2017; Wijayanto et al., 2020; Ozdemir et al., 2021), Extreme Learning Machine (Yuan et al., 2014), K-Nearest Neighbor (Guo et al., 2011; Tuncer et al., 2021), Deep Neural Network (Sayeed et al., 2019), Recurrent Neural Network (Yavuz et al., 2018).

Gotman (1982) proposed the first widely used new method, which is based on decomposing the EEG into elementary waves and detecting paroxysmal bursts of rhythmic activity with a frequency between 3 and 20 cycles per second. This method was further improved by the same group, who broke down EEG signals into half waves and then extracted features such as peak amplitude, duration, slope, and sharpness to detect seizure activities (Gotman, 1990). Jaiswal and Banka (2017) primarily used time-domain features such as local neighborhood descriptive patterns and one-dimensional local gradient patterns for epilepsy detection. Gao et al. (2020) and Wijayanto et al. (2020) extracted approximate entropy as features and combined with recurrence quantification analysis to detect epilepsy, their method achieved an accuracy of 91.75% in the Bonn dataset (Andrzejak et al., 2001). Wijayanto et al. (2020) used the Higuchi fractal dimension (HFD) to differentiate between ictal and interictal conditions in EEG signals. Many researchers focused on time domain features, while others concentrated on frequency domain, time-frequency domain, and nonlinear approaches. Altaf and Yoo (2015) combined feature extraction with classification engines, implementing multiplex bandpass filter coefficients for feature extraction. Subsequently, a nonlinear SVM was used, achieving a sensitivity of 95.1%. Kaleem et al. (2018) developed a method based on a signal-derived empirical mode decomposition (EMD) dictionary approach.

The integrated time-frequency method has been widely used for feature extraction in various approaches. For instance, Abualsaud et al. (2015) successfully detected epilepsy from compressed and noisy EEG signals using discrete wavelet transformation (DWT), achieving an accuracy of 80% when SNR = 1 dB. Feng et al. (2017) extracted features from three-level Daubechies discrete wavelet transform. Shen et al. (2017) employed a genetic algorithm to select a subset of 980 features subset and used 6 SVMs to classify EEG data into four types, i.e., normal, spike, sharp wave, and seizures. Sikdar et al. (2018) proposed a MultiFractal Detrended Fluctuation Analysis (MFDFA) to address the multifractal behaviors in healthy (Group B), interictal (Group D), and ictal (Group E) patterns. Yavuz et al. (2018) extracted mel frequency cepstral coefficients (MFCCs) as features and applied them in a regression neural network. Goksu (2018) extracted Log Energy Entropy, Norm Entropy, and Energy from wavelet packet analysis (WPA) as features and used multilayer perception (MLP) as a classifier, achieving commendable performance.

Some researchers have used nonlinear or mixed features as classification criteria. Zeng et al. (2016) extracted Sample Entropy and the permutation Entropy, and Hurst Index from EEG segments which were selected through an ANOVA test by four classifiers (Decision Tree, K-Nearest Neighbor Discriminant Analysis, SVM). Ren and Han (2019) extracted both linear and nonlinear features and classified them using an extreme learning machine. Sayeed et al. (2019) employed DWT, Hjorth parameters, statistical features, and a machine learning classifier to differentiate between ictal EEG and interictal EEG patterns.

These methods based on feature extraction are influenced by the intrinsic characteristics of EEG, such as muscle activities and eye movements, which may introduce noise to the original EEG data, potentially altering its actual characteristics (Hussein et al., 2019; Li et al., 2020). To address these challenges, many deep learning models have been developed for automatic epileptic seizure detection.

While other approaches have been proposed in the literature for epilepsy classification (Joshi et al., 2014; Zhu et al., 2014; Hassan et al., 2016; Indira and Krishna, 2021; Qaisar and Hussain, 2021), the prevailing trend involves the application of deep learning techniques (Yuan et al., 2017; Acharya et al., 2018; Tsiouris et al., 2018; Ullah et al., 2018; Covert et al., 2019; Li et al., 2020; Ozdemir et al., 2021) in this domain. However, most traditional methods have focused on specific or local features, resulting in information loss, including time domain features, frequency domain features, time-frequency domain features, and nonlinear features. Deep learning methods have demonstrated strong performance across various fields and have shown promise in epilepsy classification. Therefore, we propose combining FFT feature extraction with a deep learning algorithm.

The structure of this paper is as follows: Section 2 gives a brief overview of the dataset, outlines the proposed method, and introduces the classifier used. Section 3 presents the results and compares them with other methods. Section 4 discusses the proposed approach, while section 5 highlights the main conclusions, contributions, and potential future directions.

## 2 Materials and methods

### 2.1 Epilepsy dataset

The EEG dataset used for the epilepsy classification performance is from the University of Bonn (Andrzejak et al., 2001). This comprehensive dataset includes EEG signals from both healthy individuals and those with epilepsy, with recordings taken under various conditions such as eyes opened and closed, intracranial and extracranial potential, and interictal and ictal states. The dataset is divided into five subsets labeled as A, B, C, D, and E, each containing 100 single-channel EEG signal segments. Each signal segment is 23.6 s long and sampled at a rate of 173.61 Hz. Subsets A and B were recorded using surface EEG recordings from five healthy volunteers with eyes open and closed, respectively, follow the standard electrode placement scheme of the International 10–20 System. Subsets C, D, and E consist of intracranial recordings from five epileptic patients, with set D representing recordings from the epileptogenic zone, set C from the hippocampal formation of the opposite hemisphere, and set E exclusively containing seizure recordings. Subsets C and D correspond to epileptic interictal states, while set E captures ictal activity. Further details can be found in Table 1.

**Table 1:** Bonn epilepsy dataset.

Class	A	B	C	D	E
Description	Nonepileptic eyes opened	Nonepileptic eyes closed	Epileptic interictal, epileptogenic zone	Epileptic interictal, hippocampal	Epileptic ictal

**Figure 1:**
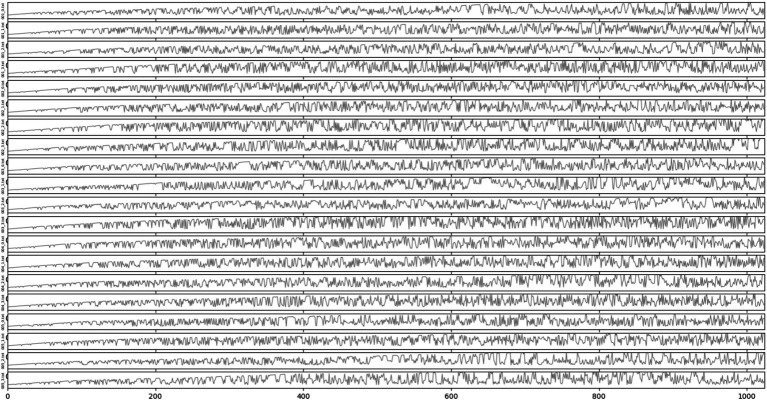
Signal display.

Each EEG set in the dataset contains 100 segments, each segment containing 4,096 points. However, since the classifier uses a CNN network, having more segments in the dataset is crucial for influencing the algorithm’s performance. To address this issue, we divide each EEG segment into four epochs, each comprising 1,024 points. As a result, the original dataset transforms into one containing five classes (A, B, C, D, and E), with 400 segments each having 1,024 sampling points (Pachori and Patidar, 2014; Figure 1).

In order to determine the performance and accuracy of the epilepsy classification algorithm, 9 classifications are considered to be designed as follows, they are A vs. E, B vs. E, AB vs. E, C vs. E, D vs. E, CD vs. E, AB vs. CD, AB vs. CDE, and ABCD vs. E.

A vs. E and B vs. E can determine if eye closure or opening influences epilepsy detection. AB vs. E, A vs. E, and B vs. E can assess the impact of additional EEG data on epilepsy detection.

C vs. E evaluates the method’s performance in distinguishing interictal from ictal patterns. D vs. E examines the method’s effectiveness in classifying interictal from ictal patterns and exploring the relationship between brain activity and hippocampal formation in the opposite hemisphere. C vs. E and D vs. E can identify which EEG component (epileptic zone or opposite hemisphere) is more effective in classifying interictal and ictal patterns. C vs. E, D vs. E, and CD vs. E investigate the influence of additional EEG data on interictal-ictal detection.

AB vs. CD tests the method’s ability to differentiate healthy volunteers from epileptic interictal patients. AB vs. CDE assesses the method’s capability to distinguish healthy volunteers from epileptic patients. ABCD vs. E evaluates the method’s capacity to differentiate seizure-free individuals from those experiencing seizures. These binary classification tasks are designed to enhance the effectiveness of the experiments.

All of these binary classification tasks are designed to enhance the effectiveness of the experiments.

### 2.2 Methods

The proposed automatic system for epilepsy classification is based on FFT feature extraction, CNN, and LSTM.

#### 2.2.1 FFT

Three approaches are selected for comparison to determine an optimal method for binary classification: FFT, wavelet transformation (WT), and EMD features. The discussion section compares the proposed methods with other approaches to assess their performance.

The widely used convolution theorem asserts that circular convolutions in the spatial domain are equivalent to pointwise products in the Fourier domain. Matrix generation plays a crucial role in the proposed framework as a means of quantitatively describing EEG records. The information contained in the EEG record matrix is influenced by fast Fourier transformation (FFT) during classification tasks. The classical FFT comprehensively describes and analyzes EEG traces in the frequency domain (Samiee et al., 2015). To effectively extract valuable features from epilepsy EEG signals, the improved method of FFT is employed to convert an EEG signal into a matrix. The steps involved are outlined below:

Step 1: obtain the Fourier coefficient for a given signal $x\left(n\right)$ in the frequency range $\left[0,2\pi \right]$ using the discrete Fourier transform algorithm. The discrete Fourier transform is defined as equation (1):


(1) $$X\left(k\right)=\overset{N-1}{\underset{n=0}{\sum }}x\left(n\right)e^{-i2\pi k\frac{n}{N}}\;0\leq k\leq N-1\text{,}$$

where $X\left(k\right)$ are the discrete Fourier transform coefficients, M is the length of the input EEG.

Step 2: calculate the absolute values of the coefficients as $A_{r}=\left|X\left(k\right)\right|$ .

Step 3: transform the $A_{k}$ into the m×n . Matrix form according to the sequential order of the sample points. The resulting matrix is then expressed as equation (2):


(2) $$X=\left[\begin{array}{c} A_{1}A_{2}\cdots A_{n}\\ A_{n+1}A_{n+2}\cdots A_{n+n}\\ \vdots \vdots \vdots \vdots \\ A_{\left(m-1\right)n+1}A_{\left(m-1\right)n+2}\cdots A_{\left(m-1\right)n+n} \end{array}\right]$$

where m and n are the matrix row and matrix column, respectively.

Extracting the FFT features is a crucial step, followed by utilizing these features as training data to train the classifier.

#### 2.2.2 DWT

Wavelets can be defined as small waves with limited duration and an average value of 0. They are mathematical functions that can localize a function or data set in both time and frequency. The concept of wavelets can be traced back to Haar’s thesis (Daubechies, 1992; Adeli et al., 2003) in 1909. The wavelet transform is a powerful tool in signal processing, known for its advantageous properties such as time-frequency localization (capturing a signal at specific time and frequency points, or extracting features at different spatial locations and scales) and multi-rate filtering (distinguishing signals with varying frequencies). By leveraging these properties, one can extract specific features from an input signal that exhibit distinct local characteristics in both time and space.

In continuous wavelet transform (CWT), the signal to be analyzed is matched and convolved with the wavelet basis function in a continuous sequence of time and frequency increments. Even in CWT, the data must be digitized. Continuous time and frequency increments mean that data at each digitized point or increment is used. Consequently, the original signal is represented as a weighted integral of the continuous basis wavelet function. In DWT, the basis wavelet function takes the original signal’s inner product at discrete points (usually dyadic to ensure orthogonality). The result is a weighted sum of a series of base functions. The wavelet transform is based on the wavelet function, a family of functions that satisfy certain conditions, such as continuity, zero mean amplitude, and finite or near-finite duration.

The CWT of a square integrable function of time, $f\left(t\right)$ , is defined as equation (3):


(3) $$CWT_{a,b}=\overset{-\infty }{\underset{+\infty }{\int }}f\left(t\right)\frac{1}{\sqrt{|a|}}\psi *\left(\frac{t-b}{a}\right)dt$$

by Chui (1992), where a,b∈R,a≠0 , R is the set of real numbers, the star symbol ‘*’ denotes the complex conjugation. In CWT, the parameters a and b are continuously varying and can have infinite number of values to be taken, but this kind of computation cannot be done in finite time for modern computers. So we take a and b as discrete according to certain rules, which is DWT. If a expands exponentially, we define a as:


$$a=a_{0}^{m}$$

Since for wide wavelets we want to translate in larger steps, we can define b as:


$$b=nb_{0}a_{0}^{m},\mathrm{where}\;b_{0}>0\;\mathrm{is}\;\mathrm{fixed}\;\mathrm{and}\;n\in Z$$

The wavelet function and the transform equation are given by the following two equations, respectively equations (4), (5):


(4) $$\psi_{m,n}\left(k\right)=a_{0}^{-\frac{m}{2}}\psi \left(a_{0}^{-m}\left(k-nb_{0}a_{0}^{m}\right)\right)$$

(5) $$DWT_{m,n}=a_{0}^{-\frac{m}{2}}\overset{\infty }{\underset{k=-\infty }{\sum }}f\left(k\right)\cdot \psi *\left(a_{0}^{-m}k-nb_{0}\right)\;m,n\in \mathbb{Z}$$

#### 2.2.3 EMD

The principle of the EMD technique is to automatically decompose a signal into a set of band-limited functions called Intrinsic Mode Functions (IMFs). Each IMF must satisfy two fundamental conditions (Huang et al., 1998; Bajaj and Pachori, 2012): (1) the number of extreme points and zero crossings in the entire dataset must either be equal or differ by at most one, and (2) the mean value of the envelopes defined by local maxima and minima must be zero at every point (Li et al., 2013).

The EMD is capable of decomposing a segment of EEG signal $x\left(n\right)$ into N IMFs: $imf_{1},imf_{2},\ldots ,\;imf_{n}$ and a residue signal r . Therefore, $x\left(n\right)$ can be reconstructed as a linear combination equation (6):


(6) $$x\left(n\right)=\overset{N}{\underset{n=1}{\sum }}imf_{n}+r$$

The following describes a systematic method for extracting IMFs:

Given an input signal


$$x\left(n\right),r\left(n\right)=x\left(n\right),n=0.$$

Step 1: determine the local maximum and local minimum of $x\left(n\right)$ .

Step 2: determine the upper envelope $e_{\text{max}}\left(n\right)$ by connecting all local maximum through cubic spline functions. Repeat the same procedure for the local minima to produce the lower envelope $e_{\text{min}}\left(n\right)$ .

Step 3: calculate the mean value for each point on the envelopes: $m\left(n\right)=\left(e_{\text{max}}\left(n\right)+e_{\text{min}}\left(n\right)\right)/2$ .

Step 4: the equation $h\left(n\right)=x\left(n\right)-m\left(n\right)$ , if $h\left(n\right)$ satisfies the IMF condition, then $n=n+1,imf_{n}=h\left(n\right)$ , go to step 5, else $x\left(n\right)=h\left(n\right)$ , cycle 1–4.

Step 5: Let $r\left(n\right)=r\left(n\right)-imf_{n}$ , if $r\left(n\right)$ is a monotonic function, end the sifting process, else, $x\left(n\right)=r\left(n\right)$ and go back to step 1.

The residue contains the lowest frequency. The main features of the ictal EEG are closely related to the first five IMFs. IMF1-IMF5 of each EEG segment is used to extract the EEG features.

#### 2.2.4 CNN + nLSTM

**Figure 2:**
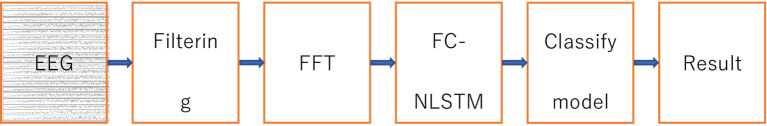
Flowchart of proposed method.

Figure 2 displays the proposed automatic system for epilepsy detection, which is based on the fully-convolutional nested long short-term memory (FC-NLSTM) model.

Each EEG signal is initially segmented into a series of EEG segments, each segment containing M sampling points, by applying a fixed-length window that slides through the entire signal. Then filter the EEG signals using a Chebyshev bandpass filter with a cutoff frequency of 3–40 Hz. These EEG segments are then inputted into a fully convolutional network (FCN) with three convolutional blocks to learn the distinctive seizure characteristics present in the EEG data. The FCN serves as a feature extractor, effectively capturing the hierarchy features and internal structure of EEG signals. Subsequently, the features learned by the FCN are inputted into the NLSTM model to uncover the inherent temporal dependencies within the EEG signals. To extract the output characteristics of all NLSTM time steps, the time-distributed fully connected (FC) layer is used to take the outputs of all NLSTM time steps as inputs, rather than just the output of the last time step. Considering that all EEG segments should contribute equally to the label classification, a one-dimensional average pooling layer is added after the time-distributed fully connected layer. Finally, an FC layer is used for classification, and a softmax layer is employed to compute the probability that the EEG segment belongs to each class and predict the class of the input EEG segment (Li et al., 2020).

Temporal convolutional networks are widely used to analyze time-series signals, enabling the capture of how EEG signals evolve and automatic learning of EEG structures from data. The raw EEG signal comprises low-frequency characteristics with long periods and high-frequency characteristics with short periods (Adeli et al., 2003). It serves as a feature extraction module in the FCN and has been demonstrated as an effective method for time-series analysis problems (Wang et al., 2017). To prevent model overfitting to noise in the training data, this study maintains simplicity and shallowness in the FCN model, which includes three stacked convolutional blocks. Each of the three basic convolutional blocks consists of a convolution layer and a Rectified Linear Unit activation function.

According to the EEG recordings that are close to or even distant from the current EEG epoch, neurologists can determine whether the EEG epoch is a part of a seizure. Recurrent neural networks have made significant progress in emulating this human ability. A more intricate model called LSTM has been proposed based on the simple recurrent neural networks, which incorporates a memory mechanism and addresses the problem of vanishing gradients (Hochreiter and Schmidhuber, 1997). This memory mechanism allows the model to retain previous information from the EEG recordings. In this study, the FC-NLSTM is used to capture the temporal dependencies in EEG signals within the output of the feature extraction module.

#### 2.2.5 Classification

The test data is inputted into the classification model for classification in this step. The 10-fold cross-validation method split the data into 10 parts, using 9 parts to train the model and reserving 1 part as the test set to evaluate the model’s performance. This process is repeated 10 times to calculate the average sensitivity, specificity, and accuracy values.

FFT, DWT, and EMD are chosen as features for training and testing, with the results compared in part 3. Subsequently, the best-performing features were selected as the method feature and compared against the performance of existing methods.

### 2.3 Classifier result estimation

All the experiments results are based on the Bonn University database. The 10-fold cross-validation is used to reduce potential system errors, as well as to assess the stability and reliability of the proposed model.

The EEG data is evenly split into 10 subsets. Nine subsets are designated as training sets, while the remaining one is assigned to test the model. This iterative process is repeated 10 times, and the averaged values across these runs are computed. The performance assessment of the proposed method involves statistical evaluation measures such as sensitivity, specificity, and recognition accuracy.

Before delving into the statistical measures of sensitivity, specificity, and recognition accuracy, let us provide descriptions of four fundamental concepts:

True positive (TP): the number of positive (abnormal) examples classified as positive.

False negative (FN): the number of positive examples classified as negative (normal).

True negative (TN): the number of negative examples classified as negative.

False positive (FP): the number of negative examples classified as positive.

Sensitivity (Sen) is calculated by dividing true positive (TP) by the total number of seizure epochs identified by the experts. TP represents the seizure epochs marked as positive by both the classifier and EEG experts.

Sen = TP/(TP + FN).

Specificity (Spe) is computed by dividing TN by the total number of non-seizure epochs identified by the experts. TN encapsulates the count of non-seizure epochs identified correctly.

Spe = TN/(TN + FP).

Accuracy (Acc) is the number of correctly marked epochs divided by the total number of epochs.

Acc = (TP + TN)/(TP + TN + FP + FN).

## 3 Results

All experiments are performed in Python using Keras with TensorFlow backend and are implemented on an NVIDIA GeForce GTX1080-Ti GPU machine. In order to fully evaluate the performance of the proposed method in ideal and real situations, the University of Bonn database is used in this study.

All 9 tasks are tested in three methods. Table 2 shows that FFT and FC-NLSTM obtained the best accuracy in all tasks except ABCD vs. E. EMD performed poorly in every task except ABCD versus E. Therefore, FFT is selected as the optimal feature for comparison with other methods in subsequent sections.

**Table 2:** Nine accuracy of three methods in different tasks.

Tasks	FFT	DWT	EMD
A vs. E	0.9962	0.8975	0.7312
B vs. E	0.9900	0.9425	0.5525
AB vs. E	0.9983	0.9658	0.7833
C vs. E	0.9913	0.9338	0.5000
D vs. E	0.9763	0.9400	0.8925
CD vs. E	0.9867	0.9533	0.6667
AB vs. CD	0.9906	0.8631	0.9569
ABCD vs. E	0.9815	0.9655	0.9915
AB vs. CDE	0.9795	0.8505	0.7890

### 3.1 Normal or interictal or non-ictal vs. ictal classification

Three types of data are used in the experiment. They include non-ictal vs. ictal(A vs. E, B vs. E, AB vs. E, C vs. E, D vs. E, CD vs. E, AB vs. CDE, ABCD vs. E), and normal vs. interictal (AB vs. CD).

The first three experiments compare non-ictal with ictal conditions, including A vs. E, B vs. E, and AB vs. E. The second set of three experiments compare non-ictal with ictal conditions including C vs. E, D vs. E, CD vs. E. The third experiment focuses on distinguishing between non-ictal and ictal states, classifying ABCD as seizure-free and E as seizure epilepsy. These experiments are conducted to validate the effectiveness and reliability of the proposed method.

Table 3 presents the results of the two-class seizure detection problem. As shown in this table, the proposed method demonstrates excellent classification performance across all normal vs. ictal scenarios, achieving nearly 100% sensitivity, specificity, and accuracy in some instances. Although not every fold in the 10-fold cross-validation reaches 100%, the mean sensitivity, specificity, and accuracy values exceed 99%. Notably, the specificity for A vs. E reaches 100%. In the interictal vs. ictal comparison, the proposed method also performs well, achieving 100% sensitivity, specificity, and accuracy in half of the folds in the 10-fold cross-validation. The highest sensitivity of 100% is achieved in the C vs. E experiment, with nearly 100% performance in terms of sensitivity, specificity, and accuracy in multiple folds for C vs. E, D vs. E, and CD vs. E. In the non-ictal vs. ictal experiments ABCD vs. E, our method achieves a mean accuracy of 98.15%. All classification results exhibit an accuracy rate above 97.63%, demonstrating the robustness of our methods across various classification tasks. Among these experiments, the highest mean accuracy of 99.83% is observed in AB vs. E. Data imbalance is evident in these experiments, with the sensitivity, specificity, and accuracy in ABCD vs. E being lower compared to other experiments. The imbalance of non-ictal data segments in ABCD vs. E is four times greater than A vs. E, B vs. E, C vs. E, D vs. E, and twice as much as AB vs. E and CD vs. E. In this case, the traditional machine learning approaches may struggle to predict the minority classes (Kundu et al., 2013; Hussein et al., 2019). However, our methods continue to perform well under these conditions, without additional operations in our experiment. The 10-fold cross-validation thoroughly validates the method and mitigates the randomness of these experiments.

**Table 3:** The results of 10-fold cross-validation for non-ictal vs. ictal based on the Bonn University database.

		*K*1	*K*2	*K*3	*K*4	*K*5	*K*6	*K*7	*K*8	*K*9	*K*10	Mean
A vs. E	Acc	1.0000	1.0000	1.0000	0.9875	0.9875	0.9875	1.0000	1.0000	1.0000	1.0000	0.9962
	Sen	1.0000	1.0000	1.0000	0.9756	0.9756	0.9756	1.0000	1.0000	1.0000	1.0000	0.9927
	Spe	1.0000	1.0000	1.0000	1.0000	1.0000	1.0000	1.0000	1.0000	1.0000	1.0000	1.0000
B vs. E	Acc	1.0000	0.9875	0.9875	0.9625	0.9875	1.0000	0.9750	1.0000	1.0000	1.0000	0.9900
	Sen	1.0000	1.0000	0.9750	1.0000	0.9750	1.0000	1.0000	1.0000	1.0000	1.0000	0.9950
	Spe	1.0000	0.9750	1.0000	0.9250	1.0000	1.0000	0.9500	1.0000	1.0000	1.0000	0.9850
AB vs. E	Acc	1.0000	1.0000	0.9917	1.0000	0.9917	1.0000	1.0000	1.0000	1.0000	1.0000	0.9983
	Sen	1.0000	1.0000	0.9750	1.0000	1.0000	1.0000	1.0000	1.0000	1.0000	1.0000	0.9975
	Spe	1.0000	1.0000	1.0000	1.0000	0.9875	1.0000	1.0000	1.0000	1.0000	1.0000	0.9988
C vs. E	Acc	1.0000	0.9750	1.0000	1.0000	0.9750	0.9875	0.9875	1.0000	1.0000	1.0000	0.9875
	Sen	1.0000	0.9750	1.0000	1.0000	1.0000	1.0000	1.0000	1.0000	1.0000	1.0000	1.0000
	Spe	1.0000	0.9750	1.0000	1.0000	0.9500	0.9750	0.9750	1.0000	1.0000	1.0000	0.9750
D vs. E	Acc	0.9625	1.0000	0.9750	1.0000	0.9375	0.9875	0.9875	0.9500	0.9750	0.9875	0.9763
	Sen	0.9750	1.0000	0.9750	1.0000	1.0000	1.0000	0.9750	1.0000	1.0000	0.9750	0.9900
	Spe	0.9500	1.0000	0.9750	1.0000	0.8750	0.9750	1.0000	0.9000	0.9500	1.0000	0.9625
CD vs. E	Acc	0.9583	0.9750	1.0000	1.0000	1.0000	0.9833	0.9833	1.0000	0.9833	0.9833	0.9867
	Sen	1.0000	1.0000	1.0000	1.0000	1.0000	0.9500	0.9750	1.0000	0.9750	0.9750	0.9875
	Spe	0.9375	0.9625	1.0000	1.0000	1.0000	1.0000	0.9875	1.0000	0.9875	0.9875	0.9863
ABCD vs. E	Acc	0.9950	0.9950	0.9850	0.9500	1.0000	0.9900	0.9550	0.9900	0.9650	0.9900	0.9815
	Sen	0.9750	0.9750	0.9500	0.8000	1.0000	0.9750	0.8000	0.9500	0.9250	0.9500	0.9300
	Spe	1.0000	1.0000	0.9938	0.9875	1.0000	0.9938	0.9938	1.0000	0.9750	1.0000	0.9944

### 3.2 Normal vs. epileptic classification

In this section, we discuss two types of epilepsy classification problems to demonstrate the effectiveness and robustness of our proposed method, which includes two experiments comparing normal vs. interictal and normal vs. interictal and ictal cases. The former experiments are AB vs. CD, while the latter compares AB vs. CDE. Table 4 presents the classification results of sensitivity, specificity, and accuracy obtained through 10-fold cross-validation. In our experiment comparing normal vs. interictal (AB vs. CD), our methods achieve mean accuracy, sensitivity and specificity of 99.06, 98.87, and 99.25%, respectively. The comparison between normal vs. interictal and ictal cases yields a mean accuracy of 97.95%, mean sensitivity of 97.58%, and mean specificity of 98.50%.

**Table 4:** Results of 10-fold cross-validation for normal vs. interictal and normal vs. interictal and ictal based on the Bonn University database.

		*K*1	*K*2	*K*3	*K*4	*K*5	*K*6	*K*7	*K*8	*K*9	*K*10	Mean
AB vs. CD	Acc	0.9812	0.9875	0.9875	1.0000	0.9938	0.9875	0.9938	1.0000	0.9938	0.9812	0.9906
	Sen	0.9875	0.9750	0.9875	1.0000	0.9875	0.9875	0.9875	1.0000	1.0000	0.9750	0.9887
	Spe	0.9750	1.0000	0.9875	1.0000	1.0000	0.9875	1.0000	1.0000	0.9875	0.9875	0.9925
AB vs. CDE	Acc	0.9800	0.9650	0.9850	0.9800	0.9900	0.9700	0.9950	0.9800	0.9750	0.9750	0.9795
	Sen	0.9833	0.9583	0.9750	0.9750	0.9833	0.9750	1.0000	0.9667	0.9667	0.9750	0.9758
	Spe	0.9750	0.9750	1.0000	0.9875	1.0000	0.9625	0.9875	1.0000	0.9875	0.9750	0.9850

Every aspect of the AB vs. CD comparison is superior to the AB vs. CDE comparison. The key to this difference lies in the use of different data. The combination of ictal and interictal segments and interictal reduces the accuracy, sensitivity and specificity. Conversely, AB vs. E (in Table 2) achieves better results than AB vs. CDE across all evaluation metrics, with accuracy at 99.67%, sensitivity at 99.27%, and specificity at 100.00%. Ictal segments are easier to detect than interictal segments, as evidenced by the superior classification results of the AB vs. E compared to AB vs. CD. These three experiments (AB vs. E, AB vs. CD, AB vs. CDE) demonstrate that ictal segments have greater discriminative power than interictal segments, and the combination of both types makes it more challenging to classify them from normal segments. The experimental results indicate that the proposed method performs well in distinguishing non-ictal from ictal segments and excels in classifying interictal vs. ictal and normal vs. interictal and ictal segments.

## 4 Discussion

In this study, the deep learning model NLSTM uses FFT as a feature to classify epilepsy segments from normal or interictal segments or a combination of both. The model demonstrates excellent accuracy, sensitivity, and specificity in the Bonn University database. The effectiveness of our approach is validated through 9 experiments presented in Table 2. FFT is employed as a feature within the model and integrated with fully convolutional deep learning and long short-term memory to differentiate between ictal and non-ictal segments. This method uses the FFT features derived from the original EEG data.

The deep learning framework model can effectively learn overall features. The low-level layers of a FCN can capture the internal structure of EEG segments and then transmit them to the higher-level layers of the model for further processing. Subsequently, these EEG features are used to extract the temporal information by being passed to the NLSTM. The NLSTM differs from standard LSTM and the stacked LSTM models in that it enhances the depth of LSTM by nesting to select pertinent information from the EEG segments. In the traditional stacked LSTM architecture, several standard LSTM units are combined into a whole, with the processing outcome of this step serving as the input for the subsequent units. Conversely, the NLSTM structure employs external memory cells to select and process EEG segments, while internal memory cells are responsible for storing and processing them. These two modules are interdependent, with the internal module using the output of the external module as input data. This configuration demonstrates strong performance in capturing the long-term dependencies present in EEG signals.

Most epilepsy detection methods typically involve the extraction or design of features by humans to characterize epilepsy EEG. Subsequently, selection algorithms are applied to identify the most representative features for classification using various classifiers. However, these methods are often complex and time-consuming due to the search for suitable features. In contrast, deep learning frameworks, such as our approach, streamline the process by bypassing feature extraction or automating it, eliminating the need for manual feature selection common in traditional methods. This approach enables the extraction of EEG segment features without human intervention, facilitating the classification of segments into ictal or non-ictal categories. Implementing this method in medical settings alleviates the workload of neurologists by simplifying EEG graph interpretation, thereby reducing the expertise threshold and saving time for healthcare professionals.

Different lengths of EEG segments significantly affect the accuracy of normal vs. interictal vs. ictal problems, which has been demonstrated by Li et al. (2020) that the EEG segment length of 1,024 allows the method to achieve optimal accuracy. This result is verified in the three databases, which include the Bonn University database, the Freiburg Hospital database, and the CHB–MIT database.

There are many methods that have shown good performance in two-class seizure recognition problems. It is necessary and important to compare the accuracy with other research results. The results are compared in Table 5, which consists of three columns containing information on tasks, methods, and the accuracy of the classification experiments. This table includes 9 experiments conducted using the Bonn University database. Our method demonstrates higher accuracy than many other methods across all experiments. Bhattacharyya et al. (2017) used the tunable-Q wavelet transform (TQWT) to extract EEG features, which were then processed using a wrapper-based feature selection method and inputted into an SVM for the identification of ictal EEGs. They achieved 100% accuracy in A vs. E and B vs. E, and 99.5% accuracy in C vs. E. From Table 5, we can see that our method has a good performance in all 9 experiments. Kaya and Ertuğrul (2018) achieved 100% accuracy in A vs. E, but did not perform well in other tasks. Li et al. (2020) achieved 100% accuracy in A vs. E, B vs. E, and CD vs. E. Sharma et al. (2017) and Tuncer et al. (2021) both achieved 100% accuracy in B vs. E. Sharma et al. (2017) also achieved the same accuracy in AB vs. E. Our method demonstrates good performance across all nine classification tasks and achieves a classification accuracy of 99.06% in AB vs. CD.

**Table 5:** Comparison results for A vs. E, B vs. E, AB vs. E, C vs. E, D vs. E, CD vs. E, AB vs. CDE, ABCD vs. E, AB vs. CD class recognition.

Task	Sample size	Method	10-fold CV	Acc (%)	Our Acc(%)	*p*-value
A vs. E	800	Siuly et al. (2018)	No	99.5	**99.62**	/
		Tuncer et al. (2021)	Yes	99.5		/
		Zhu et al. (2014)	Yes	99		/
		Kaya and Ertuğrul (2018)	Yes	100		/
		Kaya et al. (2014)	Yes	99.5		/
		Fathima et al. (2011)	No	99.75		/
		Das et al. (2016)	No	100		/
		Al Ghayab et al. (2016)	No	99.9		/
		Fu et al. (2014)	Yes	99.13		/
		Bhattacharyya et al. (2017)	Yes	100		/
		Yuan et al. (2014)	Yes	98.63		/
		Tawfik et al. (2016)	Yes	99.5		/
		Ullah et al. (2018)	Yes	99.9		0.2385
		Li et al. (2020)	Yes	100		0.0652
B vs. E	800	Siuly et al. (2018)	No	99	**99.00**	/
		Tuncer et al. (2021)	Yes	100.0		/
		Zhu et al. (2014)	Yes	97		/
		Kaya and Ertuğrul (2018)	Yes	97.5		/
		Wang et al. (2016)	Yes	95		/
		Richhariya and Tanveer (2018)	Yes	95.0		/
		Sharma et al. (2017)	Yes	100		/
		Bhattacharyya et al. (2017)	Yes	100		/
		Swami et al. (2016)	Yes	98.9		**/**
		Li et al. (2020)	Yes	100		0.0248
		Ullah et al. (2018)	Yes	99		0.9878
AB vs. E	1,200	Sharma et al. (2017)	Yes	100	**99.83**	/
		Ullah et al. (2018)	Yes	99.8		0.0477
		Li et al. (2020)	Yes	100		0.1510
C vs. E	800	Siuly et al. (2018)	No	98.5	**99.13**	/
		Tuncer et al. (2021)	Yes	100.0		/
		Zhu et al. (2014)	Yes	98		/
		Kaya and Ertuğrul (2018)	Yes	97.5		/
		Das et al. (2016)	No	100		/
		Bhattacharyya et al. (2017)	Yes	99.5		/
		Samiee et al. (2015)	No	98.5		/
		Li et al. (2020)	Yes	99.75		0.2457
		Ullah et al. (2018)	Yes	98.1		0.1832
D vs. E	800	Siuly et al. (2018)	No	97.5	**97.63**	/
		Tuncer et al. (2021)	Yes	99.0		/
		Zhu et al. (2014)	Yes	93		/
		Kaya and Ertuğrul (2018)	Yes	94.5		/
		Das et al. (2016)	No	100		/
		Nicolaou and Georgiou (2012)	No	83.13		/
		Kumar et al. (2014)	Yes	93		/
		Wang et al. (2013)	No	97.58		/
		Sharma et al. (2017)	Yes	98.5		/
		Ullah et al. (2018)	Yes	99.4		0.8077
		Li et al. (2020)	Yes	99.88		0.0035
CD vs. E	1,200	Sharmila and Geethanjali (2020)	No	98.8	**98.67**	/
		Ullah et al. (2018)	Yes	99.7		0.8850
		Li et al. (2020)	Yes	100		0.0065
ABCD vs. E	2000	Swami et al. (2016)	Yes	95.2	**98.15**	**/**
		Orhan et al. (2011)	Yes	99.6		**/**
		Das et al. (2016)	No	100		/
		Hussein et al. (2019)	Yes	100		**/**
		Li et al. (2020)	Yes	99.9		0.0071
AB vs. CD	1,600	Ullah et al. (2018)	Yes	99.8	**99.06**	0.0471
		Sharma et al. (2017)	Yes	92.5		/
		Li et al. (2020)	Yes	98.44		0.6828
AB vs. CDE	2000	Ullah et al. (2018)	Yes	99.5	**97.95**	0.8109
		Li et al. (2020)	Yes	99.65		0.0014

Bold indicates emphasis on our accuracy.

Table 6 presents the comparative results of statistical differences found in the classification tasks for various small datasets within the Bonn dataset. The performance in A vs. E, AB vs. E, C vs. E, and AB vs. CD is better, while D vs. E and AB vs. CDE show poorer results. The variation in differentiation among these small datasets is influenced by the nature of their data, with some showing greater differentiation and others showing slightly weaker differentiation.

**Table 6:** Comparison of differentiation under different datasets.

Number	Group1	Group2	p-Value
1	A vs. E	B vs. E	0.1825
2	A vs. E	AB vs. E	0.3562
3	A vs. E	C vs. E	0.3419
4	A vs. E	D vs. E	0.0091
5	A vs. E	CD vs. E	0.0581
6	A vs. E	AB vs. CD	0.0241
7	A vs. E	ABCD vs. E	0.0659
8	A vs. E	AB vs. CDE	0.0001
9	B vs. E	AB vs. E	0.0642
10	B vs. E	C vs. E	0.641
11	B vs. E	D vs. E	0.0925
12	B vs. E	CD vs. E	0.581
13	B vs. E	AB vs. CD	0.2399
14	B vs. E	ABCD vs. E	0.8928
15	B vs. E	AB vs. CDE	0.0488
16	AB vs. E	C vs. E	0.1137
17	AB vs. E	D vs. E	0.0039
18	AB vs. E	CD vs. E	0.0178
19	AB vs. E	AB vs. CD	0.0093
20	AB vs. E	ABCD vs. E	0.005
21	AB vs. E	AB vs. CDE	0
22	C vs. E	D vs. E	0.0407
23	C vs. E	CD vs. E	0.2994
24	C vs. E	AB vs. CD	0.1121
25	C vs. E	ABCD vs. E	0.6426
26	C vs. E	AB vs. CDE	0.0082
27	D vs. E	CD vs. E	0.2034
28	D vs. E	AB vs. CD	0.5532
29	D vs. E	ABCD vs. E	0.0521
30	D vs. E	AB vs. CDE	0.6553
31	CD vs. E	AB vs. CD	0.4805
32	CD vs. E	ABCD vs. E	0.4219
33	CD vs. E	AB vs. CDE	0.1848
34	AB vs. CD	ABCD vs. E	0.1499
35	AB vs. CD	AB vs. CDE	0.7563
36	ABCD vs. E	AB vs. CDE	0.0057

## 5 Conclusion

In order to promote the application of epilepsy detection in medical practice, the integration of FFT and fully convolutional NLSTM is used in classification. The time domain of the EEG signal transforms into the frequency domain using FFT methods. The data is then divided into training and testing parts, with the former being put into NLSTM to train classification model, and the other parts being put into the classification model to classify them as normal, interictal and ictal categories. Additionally, EMD and WT and FFT are employed as data processing methods to determine the most suitable type for NLSTM, with accuracy, sensitivity and specificity serving as evaluation metrics. Among the 9 experiments conducted, the FFT method yields the best results, confirming the approach as FFT and FC-NLSTM.

In the discussion section, we compare the results with other methods. Our method achieves an accuracy rate exceeding 97.00% across all experiments. The accuracies of 99.62, 99.00, 99.83, 99.13, 97.63, 98.67, 99.06, 98.15 and 97.95% are calculated for the cases A vs. E, B vs. E, AB vs. E, C vs. E, D vs. E, CD vs. E, AB vs. CD, ABCD vs. E and AB vs. CDE, respectively. The accuracy of 6 experiments exceeds 99.00%. These comparative results demonstrate the effectiveness of our method. They indicate its potential for automated epilepsy detection. Furthermore, this model and its framework can be used for EEG signal classification, which offers practical benefits in epilepsy detection. Its performance allows not only the classification of normal vs. ictal states, but also normal vs. interictal and interictal vs. ictal states.

In future work, it is advisable to consider using large datasets, such as the Freiburg hospital database and the CHB-MIT scalp EEG database, to improve the generalizability of the method and facilitate the development of a successful model. The integration of real-time applications has the potential to greatly impact clinical practice. In addition, it is recognized that deep learning approaches have difficulty providing explanations for decisions. Therefore, novel and explainable methods may need to be proposed to effectively address the epilepsy classification problem.

## Data availability statement

The original contributions presented in the study are included in the article/supplementary material, further inquiries can be directed to the corresponding author.

## Author contributions

JN: Conceptualization, Data curation, Formal analysis, Investigation, Methodology, Project administration, Resources, Software, Validation, Visualization, Writing – original draft, Writing – review & editing. HS: Conceptualization, Funding acquisition, Methodology, Supervision, Writing – review & editing. FW: Writing – review & editing.
